# Polydeoxyribonucleotide (PDRN) in Osteoarthritis: Immunomodulatory and Matrix-Supportive Potential, A2A Receptor-Associated Mechanisms, and Translational Challenges

**DOI:** 10.3390/medicina62101834

**Published:** 2026-09-23

**Authors:** Ahreum Baek

**Affiliations:** Department of Clinical Laboratory Science, Semyung University, Jecheon 27136, Republic of Korea; ahreumbaek@semyung.ac.kr; Tel.: +82-43-649-1624

**Keywords:** polydeoxyribonucleotide, osteoarthritis, adenosine A2A receptor, intra-articular therapy, chondrogenesis, translational research

## Abstract

Osteoarthritis (OA) is a multifactorial whole-joint disorder characterized by cartilage degeneration, synovial inflammation, subchondral bone remodeling, pain, and functional impairment. Polydeoxyribonucleotide (PDRN), a DNA-derived biological preparation commonly obtained from salmonid sperm, has been investigated as a potential adjunctive therapeutic candidate for OA because of its proposed anti-inflammatory, anti-apoptotic, angiogenesis-related, and tissue-protective properties. This review critically evaluates OA-specific evidence for PDRN, including cellular and mesenchymal stem cell models, intra-articular PDRN-containing interventions, and key translational limitations. In OA-related cellular models, PDRN has been associated with reduced inflammatory and catabolic mediator expression, restoration of aggrecan expression, and support for chondrogenic differentiation under inflammatory stress. However, these findings do not establish structural cartilage regeneration or disease modification *in vivo*. Limited clinical evidence suggests that PDRN-containing interventions, including hyaluronic acid (HA) plus PDRN, may improve pain and functional outcomes in knee OA; the independent contribution of PDRN in combination regimens and its long-term structural and safety effects remain uncertain. Evidence from ischemia/reperfusion models is discussed only as supporting mechanistic context for PDRN-associated modulation of inflammation and apoptosis. Future studies should standardize product characterization, establish A2A receptor dependency, evaluate safety after repeated intra-articular administration, and conduct adequately powered trials incorporating functional, imaging-based, structural, and safety outcomes.

## 1. Introduction

Polydeoxyribonucleotide (PDRN) is a DNA-derived biological preparation that has attracted increasing attention as a potential regenerative and tissue-protective therapeutic agent. PDRN is generally described as a highly purified, heterogeneous DNA-derived preparation composed of deoxyribonucleotide polymers, with reported chain lengths ranging from approximately 50 to 2000 base pairs and molecular weights ranging from 50 to 1500 kDa [1,2]. Commercially used PDRN is commonly obtained from salmonid sperm, particularly Oncorhynchus mykiss and Oncorhynchus keta, followed by purification and sterilization procedures [2,3]. However, the physicochemical characteristics of PDRN may vary according to its biological source, extraction process, purification method, and analytical approach. Therefore, the source and compositional characteristics of each PDRN preparation should be considered when interpreting and comparing experimental findings.

PDRN has traditionally been proposed to promote tissue repair by supplying purine and pyrimidine bases for nucleic acid synthesis through the salvage pathway, which may be advantageous in metabolically compromised or injured tissues [2]. In addition to this proposed metabolic role, accumulating experimental evidence suggests that PDRN exerts anti-inflammatory, anti-apoptotic, angiogenic, and regenerative effects through adenosine A2A receptor (A2AR; ADORA2A)-associated signaling [1,2]. A2AR activation has been linked to increased intracellular cyclic adenosine monophosphate (cAMP) signaling and the subsequent regulation of inflammatory responses, vascular tone, angiogenesis, and growth factor expression [2,4]. Supporting studies in non-OA experimental settings have suggested tissue-protective and repair-related effects of PDRN; however, these findings are not treated as direct evidence of efficacy in OA and are summarized separately in Table S1.

Early studies of PDRN in non-OA models have reported tissue-protective and repair-related effects in cutaneous, vascular, and inflammatory settings. In this OA-focused review, these studies are not treated as direct evidence of efficacy in OA; selected examples are summarized separately as supporting mechanistic evidence in Table S1.

In inflammatory joint disease, PDRN reduced inflammatory cytokine production and attenuated clinical and histological severity in collagen-induced arthritis through mechanisms associated with A2AR activation [5]. Moreover, intra-articular administration of hyaluronic acid combined with PDRN has shown potential clinical benefit in patients with knee osteoarthritis, although larger and more rigorously designed trials are required to clarify its efficacy, optimal administration protocol, and long-term safety [6].

Although PDRN has been investigated in diverse tissue-injury models, the present review focuses primarily on its relevance to OA, where there is a need for therapies that address inflammatory, catabolic, and impaired reparative processes. We critically summarize OA-specific cellular, preclinical, and clinical evidence regarding PDRN, including its proposed A2AR-associated mechanisms, matrix-supportive and chondrogenic effects, and intra-articular therapeutic potential. Evidence from ischemia/reperfusion models is included only as complementary mechanistic context for PDRN-associated regulation of inflammation and apoptosis. We also highlight limitations related to product characterization, evidence quality, structural outcomes, safety, and clinical translation.

### Literature Identification and Scope of This Review

This article is a focused review, not a systematic review or meta-analysis. To improve transparency, relevant English-language publications available up to August 2026 were identified through a focused PubMed search using combinations of the terms “polydeoxyribonucleotide,” “PDRN,” “osteoarthritis,” “cartilage,” “chondrogenesis,” “adenosine A2A receptor,” “intra-articular injection,” “ischemia/reperfusion,” and “neuroprotection,” supplemented by citation tracking of key articles. Original studies, clinical studies, systematic reviews/meta-analyses, and mechanistic reviews were considered. Priority was given to studies directly evaluating PDRN in OA-related cellular, animal, or clinical settings. Studies from non-OA models were included only when they informed proposed anti-inflammatory, anti-apoptotic, or tissue-protective mechanisms of PDRN. This focused approach was intended to provide a critical OA-centered synthesis rather than a formal systematic evidence assessment.

## 2. Molecular Mechanisms of PDRN via Adenosine A2A Receptor Signaling

The therapeutic effects attributed to polydeoxyribonucleotide (PDRN) have most consistently been associated with adenosine A2A receptor (A2AR; ADORA2A)-related signaling. Early studies demonstrated that PDRN enhanced the proliferation of human skin fibroblasts through adenosine receptor-dependent mechanisms, and subsequent pharmacological and preclinical studies supported the involvement of A2AR signaling in PDRN-mediated anti-inflammatory and tissue-protective responses [1,2,5]. However, PDRN is a heterogeneous DNA-derived preparation rather than a single, chemically defined receptor ligand. Accordingly, A2AR-dependent signaling should be regarded as a major mechanistic model supported by pharmacological and preclinical evidence, rather than as a fully established direct receptor-binding mechanism in all biological settings [2].

Adenosine regulates tissue homeostasis, inflammation, and cellular responses to metabolic stress through four G protein-coupled receptor subtypes: A1, A2A, A2B, and A3 receptors. A2AR is mainly coupled to the stimulatory G protein (Gs); its activation increases intracellular 3′,5′-cyclic adenosine monophosphate (cAMP), activates protein kinase A (PKA), and influences cAMP response element-binding protein (CREB)-associated transcriptional programs. A2AR signaling can modulate immune responses and vascular tone, but the contribution of this pathway to PDRN activity remains to be validated in disease- and product-specific contexts [7,8].

In experimental models, PDRN treatment has been associated with reduced production of pro-inflammatory mediators and enhanced tissue-repair responses. In a collagen-induced arthritis model, PDRN reduced inflammatory cytokine production and attenuated both clinical and histological disease severity; these effects were associated with A2AR stimulation [5]. Similarly, PDRN-related DNA polymeric molecules reduced the expression of inflammatory cytokines and chemokines in an interleukin-1β-stimulated cellular model of osteoarthritis [9]. These observations are consistent with the known anti-inflammatory function of cAMP-associated A2AR signaling. Although the cAMP/PKA/CREB axis can influence NF-κB-associated inflammatory transcription, the extent to which PDRN directly inhibits NF-κB nuclear translocation or modulates specific NF-κB p50/p65 dynamics remains dependent on cell type and experimental context. Therefore, PDRN is more appropriately described as modulating NF-κB-associated inflammatory pathways rather than universally and directly inhibiting NF-κB signaling [8,10].

PDRN may also regulate inflammatory and cell-survival responses through pathways beyond NF-κB. In an oxygen–glucose deprivation/reoxygenation neuronal model, PDRN treatment was associated with reduced inflammatory signaling and attenuation of apoptosis-related changes. The study further suggested a role for suppressor of cytokine signaling 3 (SOCS3) and the Janus kinase/signal transducer and activator of transcription (JAK/STAT) pathway in PDRN-associated neuroprotection [11]. Because the JAK/STAT cascade is an important regulator of neuroinflammation, cellular stress responses, and ischemic injury, this pathway provides a plausible mechanistic link between PDRN treatment and neuronal protection. However, the dependence of these effects on A2AR signaling should be confirmed by receptor-selective antagonism, genetic inhibition, or other mechanistically targeted approaches [12,13].

Beyond immunomodulation, A2AR-associated signaling may contribute to tissue repair by regulating vascular responses, local perfusion, angiogenesis, and the expression of pro-repair mediators, including vascular endothelial growth factor (VEGF) [2]. In an OA-related *in vitro* wound-healing model, PDRN was associated with altered angiogenesis-related factor expression, enhanced cellular migration, and wound closure [14]. Supporting observations from non-OA experimental models are summarized in Table S1. Nevertheless, the relative contributions of A2AR signaling, nucleotide salvage, extracellular nucleotide metabolism, and product-specific physicochemical properties remain to be clarified in individual disease contexts.

## 3. Supporting Mechanistic Evidence from Experimental Ischemia/Reperfusion Models

Evidence from ischemia/reperfusion models is included here as supporting mechanistic context, rather than as direct evidence of efficacy in OA. In differentiated Neuro-2a cells exposed to oxygen–glucose deprivation/reoxygenation (OGD/R), PDRN was associated with reduced lactate dehydrogenase release, reduced TUNEL positivity, decreased BAX expression, and increased BCL-2 expression [11]. The same study reported increased SOCS3 expression and reduced phosphorylation of JAK1, JAK2, STAT1, and STAT3, together with changes in inflammatory mediator expression. These observations support the hypothesis that PDRN may modulate inflammation- and apoptosis-related pathways under ischemic-like cellular stress; however, they do not establish direct A2AR dependency or clinical neuroprotective efficacy.

An *in vivo* cerebral ischemia study also reported PDRN-associated improvement in short-term memory impairment and attenuation of inflammatory responses through MAPK-related signaling [15]. Nevertheless, the relevance of these non-OA models to OA is indirect. Their principal value of this review is to illustrate potentially shared tissue-protective mechanisms, whereas the OA-specific evidence base and its clinical translation are addressed in Section 4 and Section 5.

## 4. Tissue Regeneration and Immunomodulation in Osteoarthritis

Osteoarthritis (OA) is a chronic, heterogeneous whole-joint disorder characterized by cartilage degeneration, synovial inflammation, subchondral bone remodeling, osteophyte formation, pain, and functional impairment. Contemporary research recognizes that mechanical, inflammatory, metabolic, and pain-related phenotypes may coexist and evolve during disease progression. Synovitis, subchondral bone remodeling, and osteochondral vascular and sensory alterations contribute to symptoms and structural progression, underscoring the limitations of cartilage-centered interpretations of OA [16,17,18,19,20,21,22]. This whole-joint framework is relevant when interpreting PDRN studies because changes in chondrocyte or matrix-related markers alone cannot establish clinically meaningful disease modification. Although molecular endotyping may eventually facilitate more targeted treatment selection, validated biomarkers and prospective stratified trials are still required before this approach can guide routine care [18,19].

Pro-inflammatory cytokines, particularly interleukin-1β (IL-1β), are important mediators of OA pathogenesis. IL-1β promotes inflammatory mediator production and matrix-degrading enzyme expression, suppresses cartilage matrix synthesis, and contributes to chondrocyte dysfunction and apoptosis. Accordingly, candidate interventions should be evaluated not only for anti-inflammatory or matrix-supportive effects in cellular models, but also for their effects on synovitis, cartilage integrity, subchondral bone remodeling, pain, and physical function. In parallel, although several disease-modifying OA drug (DMOAD) strategies are under investigation, no intervention has yet been conclusively shown to provide durable structural modification across heterogeneous OA populations. Therefore, emerging intra-articular interventions should be assessed using both patient-relevant symptomatic outcomes and objective structural endpoints [23,24].

In an IL-1β-stimulated human chondrocytic cell model, PDRN treatment was associated with reduced expression of pro-inflammatory cytokines and chemokines, including IL-1β, IL-6, IL-8, and CCL3 [9]. These anti-inflammatory effects were associated with modulation of Toll-like receptor (TLR)- and nucleotide-binding oligomerization domain-like receptor (NLR)-related signaling pathways. In the same experimental setting, PDRN treatment was associated with decreased expression of matrix metalloproteinase 13 (MMP-13) and restoration of aggrecan expression [9]. These findings suggest that PDRN may attenuate inflammatory and catabolic responses in chondrocyte-related models. However, because these data were obtained *in vitro*, they should not be interpreted as direct evidence of structural cartilage regeneration or disease modification in patients with established OA.

PDRN may also influence tissue-repair-related processes in an OA-associated microenvironment. In an *in vitro* OA-related wound-healing model, PDRN increased the expression of pro-angiogenic mediators, including platelet-derived growth factor (PDGF), angiopoietin-2 (ANG-2), and vascular endothelial growth factor (VEGF), while decreasing the expression of anti-angiogenic factors such as endostatin and angiostatin. PDRN treatment was also associated with enhanced cellular migration and wound closure [14]. These findings suggest a potential pro-repair effect in this experimental model; however, angiogenesis should be interpreted cautiously in OA. Synovial and osteochondral vascular changes may contribute to synovitis, pain, and structural progression; therefore, increased angiogenesis-related factors alone cannot be regarded as evidence of beneficial structural modification in the OA joint [20,21,22].

Recent evidence also indicates that PDRN may preserve chondrogenic differentiation potential under inflammatory stress. In human bone marrow-derived mesenchymal stem cells (hBMSCs) exposed to IL-1β, PDRN attenuated reductions in chondrogenic markers, including SRY-box transcription factor 9 (SOX9), collagen type II alpha 1 chain (COL2A1), and aggrecan (ACAN). PDRN treatment was further associated with increased cartilage matrix synthesis and proteoglycan deposition [25]. In the same model, PDRN reduced apoptosis-related changes, including TUNEL positivity and BAX expression, while increasing BCL-2 expression. It also altered the expression of inflammatory and matrix-regulatory factors, including cyclooxygenase-2 (COX-2), inducible nitric oxide synthase (iNOS), tumor necrosis factor-α (TNF-α), MMP-9, MMP-13, ADAMTS-4, and tissue inhibitor of metalloproteinases 1 (TIMP-1) [25]. These results suggest that PDRN may promote a more favorable chondrogenic and matrix-regulatory environment during inflammatory stress; nevertheless, validation in OA animal models and clinically relevant cartilage-repair systems is necessary.

Additional *in vitro* evidence from lipopolysaccharide-stimulated canine chondrocytes, synoviocytes, and adipose tissue-derived mesenchymal stem cells also suggested that PDRN may reduce inflammatory cytokine and collagen-degradation-marker expression while increasing ADORA2A expression [26]. The combination of PDRN and mesenchymal stem cells showed complementary anti-inflammatory effects; however, these findings were obtained in a canine cell-based model and involved a co-intervention, precluding conclusions regarding PDRN-specific efficacy or *in vivo* OA disease modification.The OA-specific preclinical evidence discussed above, including findings from in vitro chondrocyte-related, hBMSC-based, and canine OA cell models and their key limitations, is summarized in Table 1.

Notably, direct evidence from well-characterized experimental OA animal models remains limited. Future studies should determine whether the favorable cellular effects of PDRN translate into improvements in cartilage integrity, synovitis, subchondral bone remodeling, osteophyte formation, pain-related behavior, and joint function *in vivo*.

Supportive evidence from inflammatory arthritis models further suggests that PDRN can reduce cytokine production and tissue injury through mechanisms associated with adenosine A2A receptor signaling [5]. However, collagen-induced arthritis differs biologically from OA and should not be considered direct evidence of efficacy in degenerative joint disease. This study is therefore best interpreted as supporting the broader anti-inflammatory potential of PDRN rather than as OA-specific proof of concept.

Clinical evidence for PDRN/polynucleotide-containing intra-articular interventions in knee OA remains limited but has expanded beyond the initial small studies. In a prospective randomized double-blind study, Yoon et al. compared three injections of HA plus PDRN with HA alone in 30 patients with Kellgren–Lawrence grade 2–3 knee OA and reported improvements in pain and functional outcomes over 6 months [6]. In a larger randomized double-blind HA-controlled trial, a fixed co-formulation of highly purified polynucleotides plus HA was evaluated in 100 patients with knee OA; 79 patients completed 2-year follow-up, and the combination was associated with sustained improvements in knee function and pain relative to HA alone [27]. Because both trials evaluated a combination with HA, the independent contribution of PDRN/polynucleotides cannot be determined.

More recently, a double-blind, multicenter randomized controlled trial compared three weekly intra-articular injections of polynucleotide (PN) with high-molecular-weight hyaluronic acid (HA) in 60 patients with Kellgren–Lawrence grade 1–4 knee OA. At 16 weeks, PN showed comparable improvements in weight-bearing pain, other pain measures, and functional outcomes relative to HA, without significant between-group superiority. Injection-site pain and swelling were reported at similar frequencies in both groups. This study provides useful short-term comparative evidence; however, its small sample size, short follow-up, absence of structural outcomes, and use of PN rather than an identically characterized PDRN preparation limit conclusions regarding long-term disease-modifying efficacy [28].

A 2019 systematic review and meta-analysis reported possible symptomatic benefits and no major short-term safety signals for PDRN-containing interventions, but identified substantial heterogeneity in formulation, injection protocol, comparators, and follow-up [29]. A recent evidence-based review of HA monotherapy and combination strategies similarly characterized HA–PDRN/polynucleotide combinations as an emerging approach with early promise, while emphasizing that comparative benefit and longer-term safety remain unclear [30]. The absence of major safety signals in the currently available studies should not be interpreted as proof of long-term safety, especially after repeated intra-articular administration.

Across the available clinical studies, the evidence is therefore most appropriately interpreted as short- to medium-term comparative or combination-treatment evidence for pain and function, rather than confirmation of PDRN-specific long-term safety or structural disease modification. The available clinical studies, relevant evidence syntheses, treatment regimens, and key study limitations are summarized in Table 2.

Taken together, available evidence suggests that PDRN may exert anti-inflammatory, anti-apoptotic, and matrix-supportive effects relevant to OA. At present, however, PDRN should be regarded as a promising investigational adjunct rather than an established disease-modifying OA therapy. Contemporary DMOAD development highlights the need to distinguish short-term symptomatic effects from structural benefit using validated pain and functional outcomes, imaging-based cartilage and subchondral bone assessments, biomarkers of cartilage turnover and synovial inflammation, and adequate long-term safety monitoring [23,24,31].

**Table 1:** Osteoarthritis-specific preclinical evidence for polydeoxyribonucleotide (PDRN).

Study	OA-Related Model	PDRN Exposure/Regimen	Main OA-Relevant Findings	Evidence Level and Key Limitation
Baek et al., 2018, Inflammation [9]	IL-1β-stimulated human chondrocytic cells	*In vitro*; concentration/regimen not reported	Reduced IL-1β, IL-6, IL-8, and CCL3; MMP-13 decreased and aggrecan expression restored.	*In vitro*; direct OA-related cellular evidence; no whole-joint structural outcome.
Baek et al., 2018, Cell Transplantation [14]	OA-related *in vitro* wound-healing model	*In vitro*; concentration/regimen not reported	Enhanced migration/wound closure; altered PDGF, ANG-2, VEGF, endostatin, and angiostatin expression.	*In vitro*; angiogenesis is context-dependent in OA; no *in vivo* joint outcome.
Baek et al., 2024, Scientific Reports [25]	IL-1β-exposed human bone marrow-derived MSCs	*In vitro*; concentration/regimen not reported	Preserved SOX9, COL2A1, and ACAN; increased matrix/proteoglycan deposition; apoptosis-related changes attenuated.	*In vitro*; supports chondrogenic and matrix-related responses, not confirmed cartilage regeneration.
Seo et al., 2024, Journal of Veterinary Science [26]	LPS-stimulated canine chondrocytes, synoviocytes, and adipose tissue-derived MSCs (OA cell model).	*In vitro*; PDRN alone, MSCs alone, or combination; multiple concentrations assessed.	PDRN reduced inflammatory cytokine and collagen-degradation-marker expression; increased ADORA2A expression; combination with MSCs showed complementary anti-inflammatory effects.	*In vitro* canine model; no *in vivo* OA structural or functional outcome; species and co-intervention limitations.

Abbreviations: ACAN, aggrecan; ANG-2, angiopoietin-2; IL, interleukin; MMP, matrix metalloproteinase; MSCs, mesenchymal stem cells; OA, osteoarthritis; PDRN, polydeoxyribonucleotide; PDGF, platelet-derived growth factor; VEGF, vascular endothelial growth factor.

**Table 2:** Available clinical evidence and evidence syntheses on PDRN/polynucleotide-containing intra-articular interventions for knee osteoarthritis.

Study	Design/Participants	Intervention and Comparator	Regimen and Follow-Up	Outcomes and Safety	Key Limitation
Yoon et al., 2019, Annals of Rehabilitation Medicine [6]	Prospective randomized double-blind study; 30 patients; Kellgren–Lawrence grade 2–3 knee OA.	HA plus PDRN (Placentrex) versus HA alone.	Three intra-articular injections; 6-month follow-up.	Pain and functional outcomes (VAS, WOMAC, KSS) improved in the combination group; no major short-term safety signal reported.	Combination design prevents isolation of PDRN’s independent effect; small sample and short follow-up.
Stagni et al., 2021, BMC Musculoskeletal Disorders [27]	Randomized, double-blind, HA-controlled trial; 100 patients with knee OA; 79 completed 2-year follow-up.	Fixed co-formulation of highly purified polynucleotides plus HA (PNHA) versus HA alone.	Intra-articular treatment; 2-year follow-up.	Combination associated with sustained improvement in knee function and pain; no major safety concern reported in the trial.	Combination formulation prevents attribution to PDRN/polynucleotides alone; attrition at 2 years; structural disease modification not established.
Kim et al., 2023, Scientific Reports [28]	Double-blind, multicenter randomized controlled trial; 60 patients with knee OA (Kellgren–Lawrence grade 1–4).	Intra-articular polynucleotide (PN; n = 30) versus high-molecular-weight HA (n = 30).	Three injections at 1-week intervals; 16-week follow-up.	Comparable improvements in weight-bearing pain, other pain measures, and functional outcomes; no significant between-group superiority. Injection-site pain and swelling occurred at similar frequencies in both groups.	PN rather than identically characterized PDRN; small sample, short follow-up, and no structural outcome.
Kim et al., 2019, Medicine [29]	Systematic review and meta-analysis of five randomized controlled trials in knee OA.	Intra-articular PDRN/polynucleotide-containing interventions versus HA; comparators varied across trials.	Injection protocols and follow-up periods were heterogeneous (1–4 months for several reported outcomes).	Pain reduction favored PDRN at 1 and 2 months, with no significant difference at 4 months; functional outcomes and adverse events did not differ significantly between groups.	Heterogeneous formulations/protocols/comparators; limited structural and long-term safety data.
Cao et al., 2026, Orthopedic Reviews [30]	Evidence-based review of HA monotherapy and combination strategies for knee OA; 70 studies included overall.	HA combinations, including PDRN/polynucleotides, reviewed as emerging strategies.	Literature searched from January 2010 to April 2025.	HA–PDRN/polynucleotide combinations described as early promising; comparative benefits and safety remain unclear.	Not a PDRN-specific clinical trial or meta-analysis; provides contemporary context only.

Abbreviations: HA, hyaluronic acid; KSS, Knee Society Score; OA, osteoarthritis; PDRN, polydeoxyribonucleotide; PN, polynucleotide; PNHA, polynucleotide–hyaluronic acid; VAS, visual analogue scale; WOMAC, Western Ontario and McMaster Universities Osteoarthritis Index.

## 5. Translational Considerations and Evidence Gaps

The evidence base for PDRN in OA is dominated by cellular studies, with limited direct OA animal-model evidence and a small, heterogeneous body of clinical literature. This evidence hierarchy is summarized in Table 1, Table 2 and Table 3 and should be interpreted in the context of contemporary OA phenotypes/endotypes, synovitis, subchondral bone pathology, and the ongoing challenges of disease-modifying OA drug development [18,20,23,24,31]. Differences in PDRN source, purification procedures, fragment-size and molecular-weight distribution, formulation, dose, injection regimen, and comparator interventions complicate cross-study interpretation.

For clinical translation, the main priorities are to establish the independent effect of PDRN apart from HA or other co-interventions; validate proposed A2AR-associated mechanisms using receptor-selective and genetic approaches; and test repeated intra-articular administration in well-characterized OA models. Future trials should include predefined safety monitoring for local reactions, post-injection flare, effusion, infection, hypersensitivity, and potential synovial or structural effects. Because angiogenesis may be beneficial or detrimental depending on the joint compartment and disease stage, angiogenesis-related outcomes should be interpreted together with synovitis, cartilage, bone, pain, and functional outcomes.

Adequately powered multicenter trials with standardized interventions, active comparators, and long-term follow-up are needed to determine whether PDRN provides symptomatic benefit, structural benefit, or both. Patient-relevant outcomes should include pain, function, quality of life, magnetic resonance imaging or standardized radiographic measures, biomarkers, and adverse events. Transparent reporting in accordance with applicable clinical and preclinical standards will improve reproducibility and reduce translational bias [32,33,34]. The main evidence gaps and future research priorities are summarized in Table 3.

## 6. Conclusions

PDRN is a DNA-derived biological preparation with proposed anti-inflammatory, anti-apoptotic, and matrix-supportive properties. Available OA-related cellular studies suggest that PDRN may modulate inflammatory and catabolic responses and support chondrogenic differentiation under inflammatory stress. These effects have been associated with, but do not conclusively establish, A2AR-related signaling; the contributions of nucleotide salvage, extracellular nucleotide metabolism, and product-specific characteristics also require clarification.

Current evidence is insufficient to establish PDRN as a disease-modifying therapy for OA. In particular, cellular changes in inflammatory or chondrogenic markers should not be equated with structural cartilage regeneration, and the independent clinical contribution of PDRN remains uncertain in HA combination regimens. Standardized product characterization, OA-relevant animal studies, robust mechanistic validation, and adequately powered long-term trials with structural, functional, and safety outcomes are required. PDRN should therefore be considered a promising investigational adjunct for OA rather than an established regenerative treatment.

## Figures and Tables

**Table 3:** Translational evidence gaps and future research priorities for PDRN in osteoarthritis.

Domain	Current Limitation	Priority for Future Research	Key Outcomes/Reporting Elements
Product characterization	Heterogeneous or incompletely reported source, formulation, and manufacturing characteristics.	Standardized product and batch-level characterization.	Source, purification, fragment-size/molecular-weight profile, purity, DNA integrity, sterility/endotoxin testing, storage, batch consistency.
Mechanistic validation	A2AR involvement is supported mainly by pharmacological and preclinical evidence.	Receptor-selective antagonism, binding/signaling studies, and knockdown/knockout approaches.	A2AR dependency; cAMP/PKA/CREB, NF-κB-associated, and SOCS3/JAK/STAT responses.
OA preclinical evidence	Predominantly cell-based evidence; limited direct evidence from well-characterized OA animal models.	Use whole-joint OA models and assess repeated treatment.	Cartilage integrity, synovitis, subchondral bone, osteophytes, pain-related behavior, and joint function.
Clinical efficacy and safety	Combination regimens and heterogeneous protocols limit attribution; long-term safety unknown.	Adequately powered multicenter randomized trials with active comparators and long follow-up.	Pain, function, quality of life, MRI/radiography, biomarkers, local/systemic adverse events, repeated-injection safety.
Reporting rigor	Incomplete reporting can limit comparison and reproducibility.	Prospective registration and transparent clinical/preclinical reporting.	Prespecified outcomes, missing data, adverse events, randomization, blinding, sample-size justification, and sex considerations.

## Data Availability

No new data were created or analyzed in this review. Data sharing is not applicable to this article.

## Supplementary Materials

The following supporting information can be downloaded at: https://www.mdpi.com/article/10.3390/medicina62101834/s1, Table S1. Selected non-OA mechanistic evidence for PDRN (indirect relevance to osteoarthritis).
