# Supplementary material for: Polydeoxyribonucleotide (PDRN) in Osteoarthritis: Immunomodulatory and Matrix-Supportive Potential, A2A Receptor-Associated Mechanisms, and Translational Challenges
Source: Medicina (Kaunas). 2026 Sep 23;62(10):1834. doi: 10.3390/medicina62101834 (PMC13706838; doi:10.3390/medicina62101834)
Supplement: Supplementary file 1 [file medicina-62-01834-s001.zip › medicina-4537328-supplementary.pdf]

Table S1. Selected non-OA mechanistic evidence for PDRN (indirect relevance to osteoarthritis).

**Study**	**Model**	**Key observation**	**Relevance / limitation for OA review**
Jo et al., 2023, Scientific Reports [11]	Differentiated Neuro-2a cells exposed to OGD/R.	Reduced cytotoxicity and apoptosis-related changes; SOCS3/JAK/STAT-associated signaling changes.	Supporting context for inflammation/apoptosis; not OA evidence.
Ko et al., 2021, PLoS One [15]	Cerebral ischemia model.	Improved short-term memory impairment and reduced inflammation; MAPK-related signaling.	Supporting context only; distinct tissue and disease model.
Bitto et al., 2011, Arthritis & Rheumatism [5]	Collagen-induced arthritis model.	Reduced cytokine production and arthritis severity; A2AR-associated effects.	Inflammatory arthritis is biologically distinct from OA.
